# Research on Quantitative Analysis Method of Infrared Spectroscopy for Coal Mine Gases

**DOI:** 10.3390/molecules30143040

**Published:** 2025-07-20

**Authors:** Feng Zhang, Yuchen Zhu, Lin Li, Suping Zhao, Xiaoyan Zhang, Chaobo Chen

**Affiliations:** Electronic Information Engineering, Xi’an Technological University, Xi’an 710021, China; zhang_feng@xatu.edu.cn (F.Z.); zyc774757283@163.com (Y.Z.); linli0624@xatu.edu.cn (L.L.); zhaosuping@xatu.edu.cn (S.Z.); zhangxiaoyan@xatu.edu.cn (X.Z.)

**Keywords:** FTIR, coal mine gases, baseline correction, variables selection, quantitative analysis

## Abstract

Accurate and reliable detection of coal mine gases is the key to ensuring the safe service of coal mine production. Fourier Transform Infrared (FTIR) spectroscopy, due to its high sensitivity, non-destructive nature, and potential for online monitoring, has emerged as a key technique in gas detection. However, the complex underground environment often causes baseline drift in IR spectra. Furthermore, the variety of gas species and uneven distribution of concentrations make it difficult to achieve precise and reliable online analysis using existing quantitative methods. This paper aims to perform a quantitative analysis of coal mine gases by FTIR. It utilized the adaptive smoothness parameter penalized least squares method to correct the drifted spectra. Subsequently, based on the infrared spectral distribution characteristics of coal mine gases, they could be classified into gases with mutually distinct absorption peaks and gases with overlapping absorption peaks. For gases with distinct absorption peaks, three spectral lines, including the absorption peak and its adjacent troughs, were selected for quantitative analysis. Spline fitting, polynomial fitting, and other curve fitting methods are used to establish a functional relationship between characteristic parameters and gas concentration. For gases with overlapping absorption peaks, a wavelength selection method bassed on the impact values of variables and population analysis was applied to select variables from the spectral data. The selected variables were then used as input features for building a model with a backpropagation (BP) neural network. Finally, the proposed method was validated using standard gases. Experimental results show detection limits of 0.5 ppm for CH_4_, 1 ppm for C_2_H_6_, 0.5 ppm for C_3_H_8_, 0.5 ppm for n-C_4_H_10_, 0.5 ppm for i-C_4_H_10_, 0.5 ppm for C_2_H_4_, 0.2 ppm for C_2_H_2_, 0.5 ppm for C_3_H_6_, 1 ppm for CO, 0.5 ppm for CO_2_, and 0.1 ppm for SF_6_, with quantification limits below 10 ppm for all gases. Experimental results show that the absolute error is less than 0.3% of the full scale (F.S.) and the relative error is within 10%. These results demonstrate that the proposed infrared spectral quantitative analysis method can effectively analyze mine gases and achieve good predictive performance.

## 1. Introduction

Gas monitoring plays a critical role in early warning and emergency responses to coal mine disasters. Previous studies have demonstrated that the concentration and generation rate of gases such as carbon monoxide (CO), carbon dioxide (CO_2_), methane (CH_4_), ethane (C_2_H_6_), propane (C_3_H_8_), n-butane (n-C_4_H_10_), ethylene (C_2_H_4_), acetylene (C_2_H_2_), and propylene (C_3_H_6_) can be used to evaluate the progression of coal spontaneous combustion. Following disaster events, particularly during the management and reopening of sealed mine sections, the monitoring of CO, C_2_H_4_, and C_2_H_2_ is essential to assess underground safety conditions. Additionally, toxic gases such as CO, C_2_H_4_, and C_2_H_6_ are commonly released after gas explosions. Sulfur hexafluoride (SF_6_) is frequently employed as a tracer gas to assess ventilation efficacy in underground environments. Taken together, the characteristic gases closely associated with coal spontaneous combustion, gas explosions, and ventilation diagnostics include CO, CO_2_, C_2_H_4_, C_2_H_2_, C_3_H_6_, SF_6_, CH_4_, C_2_H_6_, C_3_H_8_, n-C_4_H_10_, and iso-butane (i-C_4_H_10_) [1].

In actual coal mine environments, the concentrations of these gases can vary widely. For instance, CH_4_ may be present in ranges from 0 to 200,000 ppm, while CO ranges from 0 ppm to 2000 ppm, and CO_2_ can vary from 0 ppm to as high as 100,000 ppm. Hydrocarbons such as C_2_H_6_, C_3_H_8_, n-C_4_H_10_, and i-C_4_H_10_ typically range from 0 to 5000 ppm, and unsaturated hydrocarbons including C_2_H_4_, C_2_H_2_, and C_3_H_6_ are generally observed in the 0 to 3000 ppm range. SF_6_, used as a tracer, typically ranges up to 2000 ppm. These gases often exhibit spatial and temporal uneven distribution due to factors such as coal seam gas content, geological structure, ventilation flow patterns, mining activities, and spontaneous combustion zones. The uneven distribution and dynamic concentration changes pose significant challenges for real-time monitoring and risk assessment.

Therefore, accurate and reliable analysis of the composition and concentration of these gases is essential for the timely identification of potential safety hazards. It enables early intervention and provides critical data for explosion risk evaluation and emergency rescue planning. This predictive capability is crucial for preventing secondary disasters and holds substantial theoretical and practical value for ensuring coal mine safety [2,3,4,5,6,7].

Fourier Transform Infrared (FTIR) spectroscopy is widely recognized for its advantages, including high-speed detection, multi-component analysis, and non-destructive measurement capabilities. These features make it a powerful tool for qualitative and quantitative analysis in various fields, such as pharmaceuticals, chemical engineering, biotechnology, and environmental monitoring [8,9,10,11,12,13,14,15]. In recent years, extensive research has been conducted on infrared spectral analysis. Goldschmidt et al. [16] applied artificial neural networks to quantitatively determine N_2_O and CO, achieving coefficients of determination (R^2^) of 0.99997 and 0.99987, respectively. Our research group previously analyzed coal mine gases [17]. Firstly, a Tikhonov regularization was applied to select the spectral variables, and then models were established using both backpropagation (BP) neural networks and multiple linear regression. Furthermore, an information fusion approach was employed to predict the concentrations of five gases: CH_4_, C_2_H_6_, C_3_H_8_, i-C_4_H_10_, and n-C_4_H_10_ [14]. Shoukat et al. [18] applied PLS regression and demonstrated that FTIR can be effectively used for the simultaneous analysis of compounds such as MDEA, MEG/TEG, H_2_O, and CO_2_ in acid gas and water removal systems. Li et al. addressed the challenge of overlapping alkane spectra by introducing an adaptive moving window PLS modeling method for quantitative analysis [19].

Despite these advancements, field trials in coal mines reveal that the application of FTIR for online gas analysis still faces several critical challenges. First, the underground environment is highly variable and harsh, often leading to baseline drift in spectral signals due to prolonged exposure of spectrometers to environmental interference. Second, the diversity of gas types, uneven concentration distributions, and spectral overlaps contribute to reduced accuracy and reliability of conventional analysis models.

To address these issues, this study focuses on seven representative coal mine gases. Spectral data were collected using FTIR spectrometry under simulated underground conditions. An adaptive penalty parameter method was employed for baseline drift correction. Based on the spectral distribution characteristics, gases are categorized into two types: those with distinct absorption peaks and those with severely overlapping peaks. For the former, three spectral lines (the absorption peak and adjacent troughs) were selected for curve fitting using spline or polynomial models to establish concentration relationships. For the latter, a flexible shrinkage variable selection strategy based on frequency and regression coefficient analysis was used. These selected features served as inputs to a BP neural network for accurate prediction.

## 2. Materials and Methods

For quantitative calibration, certified standard gas mixtures were provided by Dalian Special Gases Co., Ltd. (Dalian, China). The calibration gases included CH_4_ (0–200,000 ppm), CO (0–2000 ppm), CO_2_ (0–100,000 ppm), C_2_H_6_, C_3_H_8_, n-C_4_H_10_, i-C_4_H_10_ (0–5000 ppm), C_2_H_4_, C_2_H_2_, C_3_H_6_ (0–3000 ppm), and SF_6_ (0–2000 ppm), with high-purity nitrogen as the balance gas. All standard mixtures were traceable to national primary standards and accompanied by certificates indicating a concentration uncertainty within ±2%. Furthermore, the indication error of the FTIR analyzer was evaluated using data obtained from two coal mine field sites, namely Liujia Coal Mine and Fengshuigou Coal Mine, both located in Chifeng City, Inner Mongolia, China.

The FTIR spectrometer used for analyzing coal mine gases is the Spectrum Two, manufactured by PerkinElmer. The infrared source used in the instrument is a silicon carbide (SiC) rod. And the complete radiation power was measured with a standard deuterated triglycine sulfate (DTGS) detector. The spectrometer was configured with the following parameters: The length of the gas cell is 10 cm. The spectral resolution was set as 1 cm^−1^, the spectral range was set as 400–4000 cm^−1^, and the absorbance scanning type was selected. To minimize random noise interference, the number of scans per sample was set at 8. Due to its favorable linearity, the selected apodization function was the Norton–Beer medium function.

**Figure 1:**
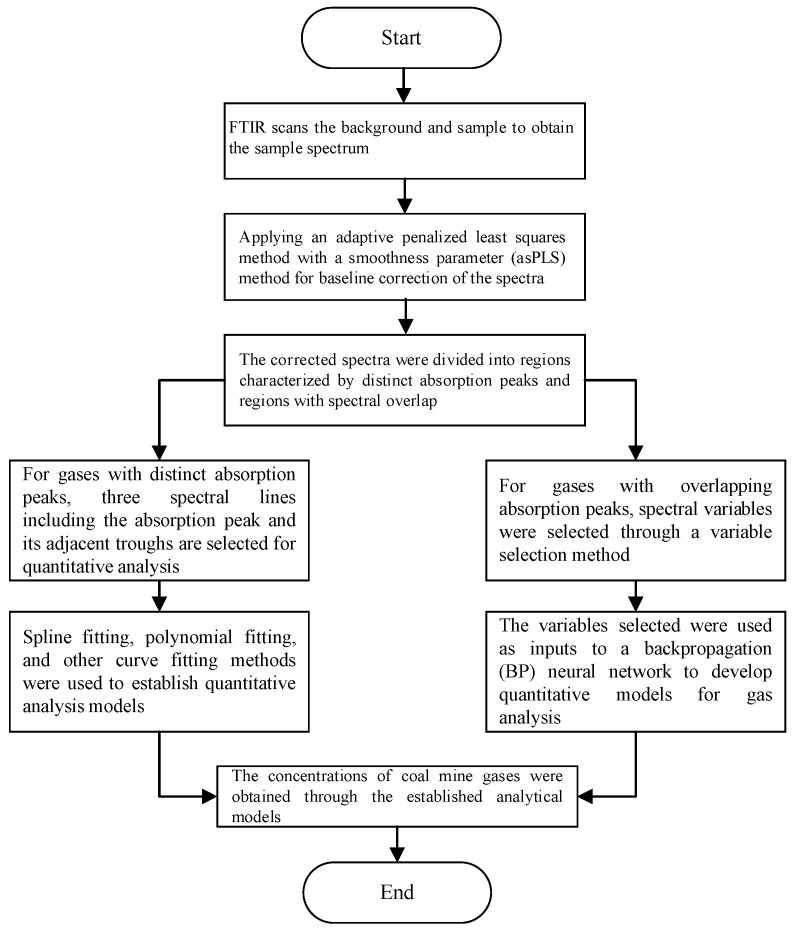
The quantitative analysis method for coal mine gases.

For multi-component gas analysis, analytical models for each gas must be established using calibration samples prepared by analytical instruments. Initially, an FTIR spectrometer was employed to acquire data on coal mine gases, and the spectral data was subsequently corrected for baseline shifts. Subsequently, based on the distribution pattern of coal mine gas, the gases are categorized into distinct absorption peak intervals and spectral overlapping absorption peak intervals. For gases with distinct absorption peaks, three spectral lines, including the absorption peak and its adjacent troughs, were selected for quantitative analysis. For the overlapping absorption regions, characteristic variables were extracted. Subsequently, a quantitative analysis model was established for each target gas. Finally, standard gases with known concentrations were used to verify the accuracy of the quantitative analysis models. An overview of the infrared spectroscopy-based quantitative analysis framework for coal mine gases is presented in Figure 1.

### 2.1. Baseline Drift Correction

**Figure 2:**
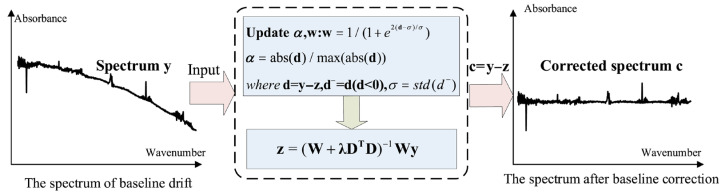
The flow chart of baseline correction by the proposed asPLS.

Spectral baseline drift is a common issue in gas analysis using Fourier Transform Infrared (FTIR) spectrometer, primarily caused by environmental variations during spectral acquisition. For example, fluctuations in the temperature of the infrared light source can lead to baseline shifts of varying magnitude. Likewise, angular deviations of the moving mirror during interferometric scanning may introduce baseline distortions in the resulting interferogram. Such baseline variations alter the absorbance values of specific spectral features, which serve as critical parameters in the quantitative analysis of gas mixtures. Consequently, uncorrected baseline drift can result in significant inaccuracies or even erroneous concentration estimations. Therefore, accurate baseline correction and identification of distorted spectra are essential to ensure the reliability of the analytical results. In this study, an adaptive penalized least squares method with a smoothness parameter (asPLS) is applied correct baseline drift in the absorption spectra of coal mine gases [20]. The baseline correction procedure is illustrated in Figure 2.

In Figure 2, w represents the weight vector. The matrix **W** = diag (w) is a diagonal matrix with the elements of w on its diagonal. **D** denotes a second-order difference matrix, and the vector d^−^ consists of the negative components of the differences between **y** and **z**. The parameter λ (lambda) is introduced to balance the trade-off between data fidelity and smoothness; **y** represents the original spectral data, containing N spectral variables.

### 2.2. Spectral Variables Selection Method

**Figure 3:**
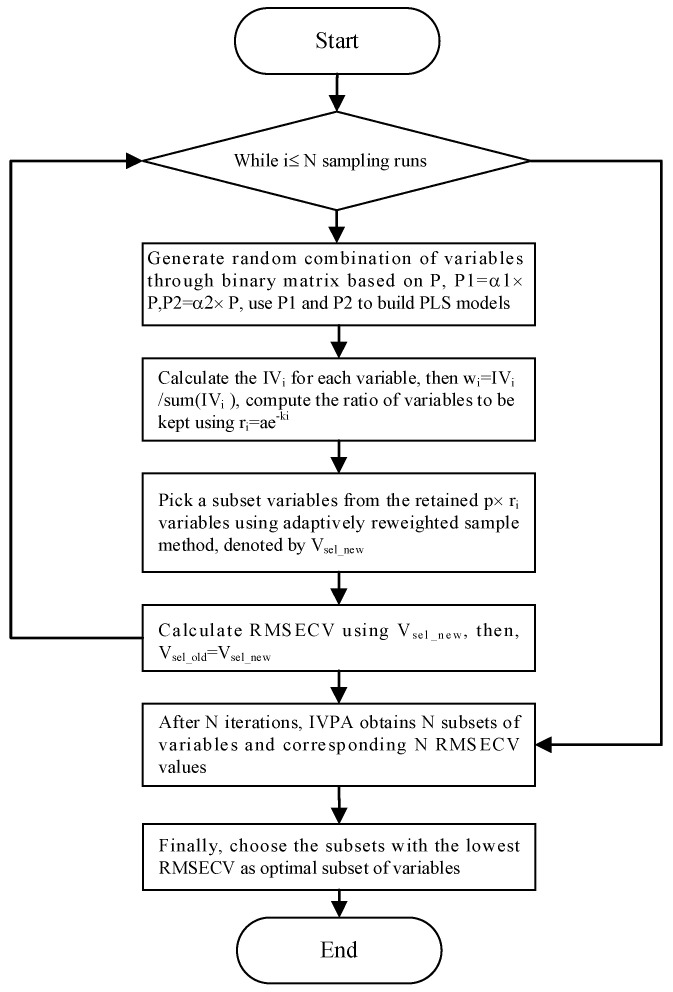
Flow chart of IVPA algorithm.

Infrared spectral data typically comprise numerous variables, some of which are irrelevant or may interfere with accurate analysis. For gases with distinct absorption peaks, satisfactory results can often be achieved by selecting a single spectral line corresponding to the main absorption peak. Linear or nonlinear regression techniques can then be applied for quantitative analysis. However, when there is significant overlap in the absorption spectra between gases, randomly selecting a few variables not only increases cross-sensitivity to other components but may also reduce the prediction accuracy for the target component. Therefore, spectral variable feature selection is crucial for reducing model computation time, improving prediction accuracy, and minimizing cross-sensitivity to other components. Here, spectral variables represent absorbance values at specific wavenumbers. In this study, based on the infrared spectral distribution characteristics of coal mine gases, the entire absorption spectrum was divided into regions of distinct absorption peaks and overlapping absorption peaks. In the overlapping absorption regions, variable selection was performed using a wavelength selection method based on the Impact Value of Variables and Population Analysis (IVPA) [21]. The schematic diagram of the IVPA algorithm is presented in Figure 3.

The spectral data obtained by scanning is denoted as matrix **X** (n × p), where n is the number of samples and p is the number of spectral lines. **Y** (n × m) is the analyte concentration information corresponding to n samples. The infrared quantitative analysis model established by PLS can be expressed as follows:

(1) y=Xβ+e

In the aforementioned formula, **β** denotes the regression coefficient vector defined as **β** = [β_1_, β_2_, …, βₚ]ᵀ, where **y** represents the concentration vector of the i-th target component, and **e** represents the random error vector. To evaluate the influence of each variable, the process begins by multiplying one of the original variables by a coefficient a_1_ (a_1_ < 1) to generate a new variable V_1_, followed by multiplying it by a coefficient a_2_ (a_2_ > 1) to obtain another variable V_2_. Partial least squares (PLS) models are then established for both V_1_ and V_2_, and the corresponding root mean square errors $UE_{i}$ and $DE_{i}$ are calculated. This procedure is repeated p times for each variable. The influence value of each variable is then computed according to Equation (2). After N sampling runs, the IVPA obtains N subsets of variables and finally chooses the subset of the lowest root mean square error of cross-validation (RMSECV) value as the optimal one.

(2) $$IV_{i}=UE_{i}-DE_{i}$$

### 2.3. Model Analysis

Based on the infrared spectral absorption characteristics of coal mine gases, the entire spectrum is divided into regions with distinct absorption peaks and gases exhibiting spectral overlap. For the target gas with a distinct absorption peak, three spectral lines at the distinct absorption peak and trough are selected for quantitative analysis, and the function between characteristic quantity and concentration is obtained by spline fitting and polynomial curve fitting methods. Numerous advanced approaches have been developed for analyzing severely overlapped absorption spectra, including partial least squares (PLS) [22], Self-Modeling Curve Resolution (SMCR) [23], Support Vector Machines (SVMs) [24], and neural networks (NNs) [25]. In this study, the NN with two layers of nodes is employed as the analysis model for each analyte component. The wavelength variables selected in Section 2.2 are used as inputs to the backpropagation (BP) neural network, and the output corresponds to the concentration of the target coal mine gas.

### 2.4. Experiment

The experimental protocol is as follows:

Step 1, preheat the infrared spectrometer for 30 min prior to spectral scanning to allow the light source temperature to stabilize.

Step 2, introduce high-purity nitrogen (99.999%) into the gas cell for approximately 3 min. During this time, perform a number of scans to obtain the background spectrum. When the background spectrum shows minimal variation after two consecutive scans, stop the background scanning and save the background spectrum data to the database.

Step 3, inject the standard gas to be detected into the gas cell, and keep the gas flow rate at 1.5 L/min by adjusting the knob of the pressure-reducing valve. After waiting 1 min, click the Scan Sample button in the analysis software(spectrum two, 10.4 version). After waiting for 20 s, the analysis software completes a measurement and displays the result on the main interface.

**Figure 4:**
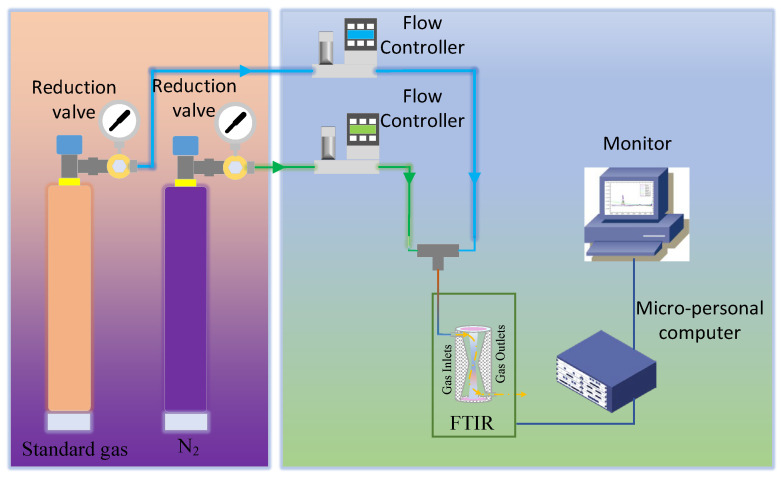
Flow chart of coal mine gas analysis by FTIR spectroscopy absorption.

Step 4, record the analysis results of each measurement, repeat 10 sets of scanning for standard gas, and calculate the repeatability of the analyzer with the analysis results of 10 sets of data. The target gases include the 11 kinds of gases (CO, CO_2_, C_2_H_4_, C_2_H_2_,s C_3_H_6_, SF_6_, CH_4_, C_2_H_6_, C_3_H_8_, n-C_4_H_10_, and i-C_4_H_10_). Each gas concentration is set to 100 ppm, 200 ppm, 500 ppm, 1000 ppm, 2000 ppm, 5000 ppm, 10,000 ppm, 20,000 ppm, 50,000 ppm, 70,000 ppm, 100,000 ppm, 200,000 ppm, and 300,000 ppm, according to the detection limit requirements. The experimental procedure is illustrated in Figure 4.

## 3. Results and Discussion

### 3.1. Spectral Baseline Drift Correction

**Figure 5:**
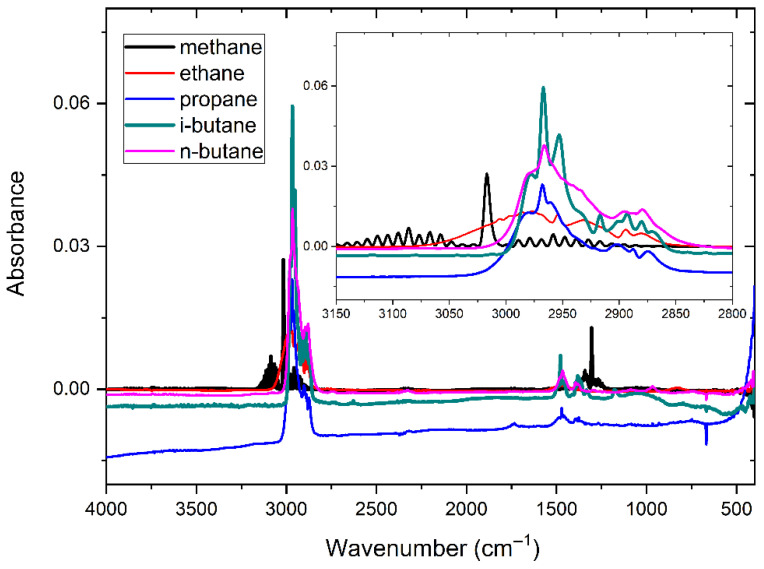
Absorption spectra of five alkane gases with a concentrssation of 500 ppm.

Figure 5 displays the absorbance spectra of five alkane components at 500 ppm: CH_4_, C_2_H_6_, C_3_H_8_, i-C_4_H_10_, and n-C_4_H_10_. It is evident that the absorption peaks of the five gases are highest near the wavenumber of 3000 cm^−1^, which is the main absorption peak of alkane gases, and in the range of 1200–1700 cm^−1^, the secondary absorption peak of alkane gases can be found. Significant spectral overlap among the five gases can be observed in both the primary and secondary absorption regions.

**Figure 6:**
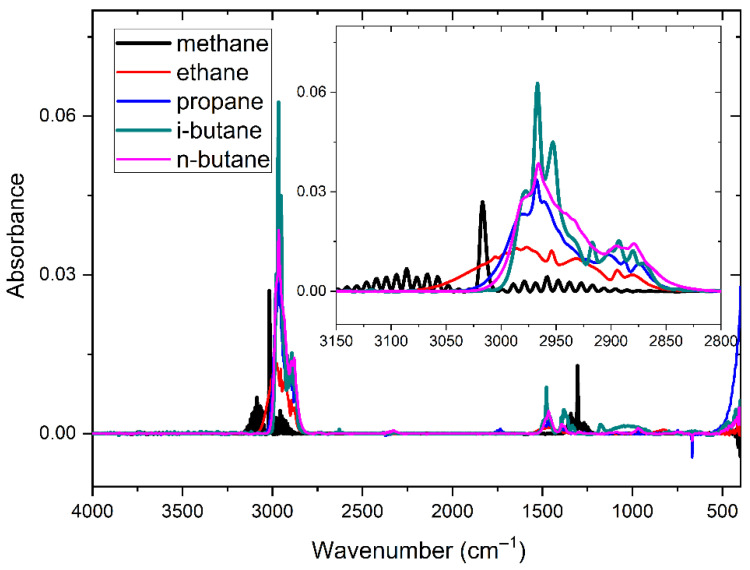
Absorbance spectra of five gases after baseline correction at 500 ppm.

The spectral baselines of the five gases displayed in the figure above exhibit varying degrees of shift. Based on previous research findings [16], spectral data underwent baseline correction utilizing a penalized least squares approach, resulting in the presentation of the baseline-corrected spectra in Figure 6.

### 3.2. Results of Characteristic Variables Extraction

**Figure 7:**
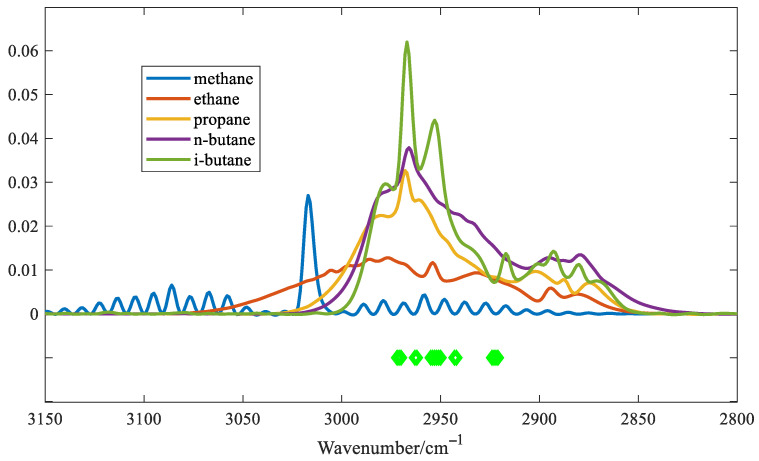
The variables selected by IVPA.

In order to evaluate the performance of the proposed algorithm, the variables selected for the IVPA method are shown in Figure 7.

The light green dots indicate the selected variables. As shown in Figure 7, IVPA selects fewer variables, and the selected variables cover the main region of the absorption peak of i-C_4_H_10_. The spectra of five alkane gases at a concentration of 500 ppm were used as input variables. A predictive model was established to estimate the concentrations of the five gases based on the variables selected by the IVPA method. The prediction results are shown in Table 1. The table indicates that IVPA shows the highest cross-sensitivity to n-C_4_H_10_, which is attributed to the similar molecular structures of n-C_4_H_10_ and i-C_4_H_10_, resulting in similar absorption spectral shapes. Conversely, the cross-sensitivity to CH_4_ is relatively low, primarily because methane exhibits significantly weaker absorbance in the wavenumber range below 3000 cm^−1^, where the variables selected for the IVPA method are concentrated. It is worth noting that IVPA has the most accurate prediction for i-C_4_H_10_, with a maximum cross-sensitivity of 1.02% and a minimum cross-sensitivity of 0.11% for the other four gases. These results indicate that this method can effectively extract and analyze variables with significant spectral overlap.

### 3.3. Quantitative Analysis Model of Gases with Distinct Absorption Peaks

**Figure 8:**
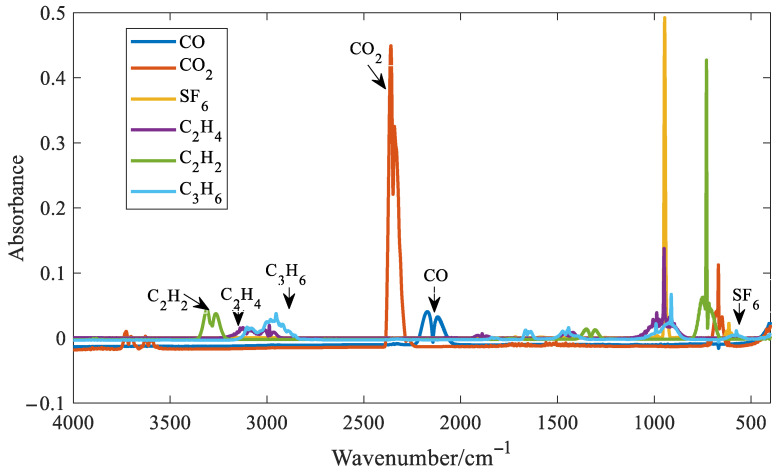
Infrared spectra of six gases.

For gases with distinct absorption peaks, such as CO, CO_2_, SF_6_, C_2_H_4_, C_2_H_2_, and C_3_H_6_, the distinct absorption peaks and troughs were selected for quantitative analysis. The absorbance spectra of the six gases are shown in Figure 8. In the figure, the concentration of SF_6_ is 500 ppm, the concentration of CO is 7000 ppm, the concentration of CO_2_ is 5000 ppm, and the concentration of the other three gases is 3000 ppm.

**Figure 9:**
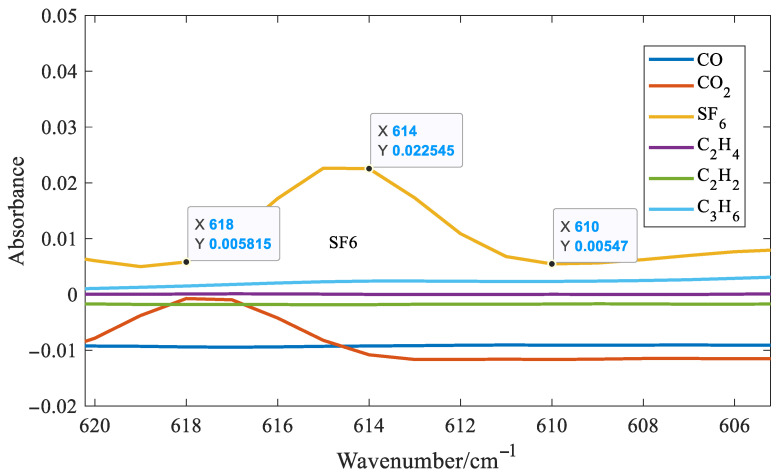
Three spectral points around the distinct absorption peak of SF_6_ at 614 cm^−1^.

It can be seen from Figure 8 that distinct absorption peaks are present for all six gases. It can also be observed that SF_6_ exhibits the highest absorbance around 1000 cm^−1^. At the same concentration, its absorption coefficient is approximately one order of magnitude higher than those of the other five gases. As a result, SF_6_ has the lowest detection limit among the six gases analyzed. Although SF_6_ shows strong absorbance near 1000 cm^−1^, this region suffers from significant spectral overlap with C_2_H_4_ and C_3_H_6_, making it unsuitable for accurate feature extraction. Fortunately, SF_6_ also presents a distinct but weak absorption peak at 614 cm^−1^. Therefore, the absorbance at this wavenumber was chosen as the characteristic variable for SF_6_ in the quantitative model. Taking SF_6_ as an example, six quantitative methods for gases with distinct absorption peaks were introduced. Three spectral points around the distinct absorption peak of SF_6_ at 614 cm^−1^ were selected, as shown in Figure 9.

As can be seen from Figure 9, the selected three spectral lines are exactly equally spaced. Therefore, the characteristic variable for SF_6_, denoted as $f_{{\mathrm{SF}}_{6}}$, is defined as follows:

(3) $$f_{{\mathrm{SF}}_{6}}=(A_{614}-A_{618})+(A_{614}-A_{610})$$

In Equation (3), A denotes the absorbance, and the subscript indicates the wavenumber; for example, A_614_ refers to the absorbance at 614 cm^−1^. As shown in Equation (3), the selected characteristic variable is not affected by baseline shifts, such as horizontal translation or linear tilt, thereby enhancing the stability and robustness of the system. This is because the distances between the three selected spectral lines are equal. When the baseline moves or tilts, the first term and the second term in Equation (3) have opposite signs and equal amplitudes, which cancel each other out. If the distances between the three selected spectral lines are not equal, the selected spectral lines are 610, 614, and 616 cm^−1^, respectively. The following expression can be constructed:

(4) $$f_{{\mathrm{SF}}_{6}}=(A_{614}-A_{610})+2\times (A_{614}-A_{616})$$

To verify the performance of the eigenvariables selected for SF_6_, the spectra of 11 target gases (CH_4_, C_2_H_6_, C_3_H_8_, n-C_4_H_10_, i-C_4_H_10_, CO, CO_2_, C_2_H_4_, C_2_H_2_, C_3_H_6_, SF_6_) with a concentration of 500 ppm were substituted into Equation (4), and the resulting characteristic value vector is [−0.00024, −0.00009, −0.00001, −0.00017, −0.00004, 0.00033, −0.00022, −0.00025, 0.00004, −0.00016, 0.0676]. It can be seen that the characteristic variable of SF_6_ has the highest sensitivity to CO, which is 0.49%. The maximum cross-sensitivity of SF_6_ to the other 10 gases is less than 0.5%, which indicates that the cross-sensitivity of the selected characteristic quantity to other gases is low, so this characteristic quantity can be selected to analyze SF_6_. Meanwhile, the spectra of ten single-component SF_6_ with volume percentage concentrations of 5 ppm, 10 ppm, 20 ppm, 50 ppm, 100 ppm, 200 ppm, 500 ppm, 1000 ppm, 2000 ppm, and 5000 ppm were substituted into Equation (4), and the corresponding eigenvalue vectors were obtained as 0.00085, 0.0017, 0.0034, 0.0082, 0.0160, 0.0307, 0.0676, 0.1127, 0.1811, and 0.2246. At this point, curve fitting methods such as spline fitting, polynomial fitting, etc., could be used to obtain the function relationship between the characteristic quantity and concentration. The fitting results obtained using the three different methods are presented in Table 2.

Table 2 presents the fitting results between the characteristic quantities and SF_6_ concentrations obtained using three different methods. It can be seen that both the quartic polynomial and cubic spline functions provide good fitting performance, while the results from quadratic polynomial fitting are comparatively less accurate. Therefore, the quartic polynomial was selected for the quantitative analysis of SF_6_, and its polynomial expression is given as follows:

(5) $$c_{{\mathrm{sf}}_{6}}=80.75f_{{\mathrm{sf}}_{6}}^{4}-24.38f_{{\mathrm{sf}}_{6}}^{3}+5.168f_{{\mathrm{sf}}_{6}}^{2}+0.4881f_{{\mathrm{sf}}_{6}}+0.0003962$$

The scanned sample spectrum was first used in Equation (4) to extract the characteristic value $f_{{\mathrm{SF}}_{6}}$, which was then input into Equation (5) to predict the SF_6_ concentration. In addition, in the actual analysis, the corresponding concentration information could be obtained according to the characteristic quantity of each gas, and then the obtained characteristic quantity was subtracted from the product of cross-sensitivity and gas concentration (i.e., the compensation method), and then the compensated characteristic quantity was substituted into Equation (5), which could obtain a more accurate analysis result. The selected spectral lines for each gas are summarized in Table 3.

The repeatability test results for gases with distinct absorption peasks are shown in Table 4.

### 3.4. Quantitative Analysis of Gases with Severe Spectral Overlap

For gas mixtures with severely overlapping absorption spectra, such as alkanes, including CH_4_, C_2_H_6_, C_3_H_8_, i-C_4_H_10_, and C_4_H_10_, the spectral data were first subjected to feature selection using the variable selection method described in Section 2.2. Subsequently, the selected features were used to develop predictive models using four different algorithms: partial least squares (PLS), backpropagation neural network (BPNN), Support Vector Machine (SVM), and Least Squares Support Vector Machine (LSSVM). Among the models, the BP neural network model demonstrated the best predictive performance. The reproducibility test results for spectral overlap are shown in Table 5.

As can be seen from Table 4 and Table 5, when the gas concentration is low, the relative standard deviation value obtained by the FTIR spectrometer is relatively large; for example, when the concentration of C_2_H_2_ is 5 ppm in the standard gas, the calculated relative standard deviation reaches 3.54%. This is because when the gas concentration is low, the average concentration of the gas obtained will also be very low, and the relative standard deviation is the ratio of the standard deviation to the average value, so the relative standard deviation will be larger when the concentration of the gas is measured at a lower level. The relative standard deviation tends to be larger for gases present at lower concentrations. It can also be seen that the standard deviations of the 10 gases are less than 10 ppm except for CO_2_. The standard deviation for CO_2_ is 77.07 ppm. This is because the standard gas has the highest concentration of CO_2_ at 20,000 ppm. The value of 77.07 ppm is very small in relation to 20,000 ppm, and the relative standard deviation of CO_2_ is only 0.36%. Thus, these test results indicate the excellent repeatability performance of the analyzer. In addition, the errors in the indicated values of the 11 characteristic gases are calculated, and it can be seen that the quoted errors of the 11 characteristic gases are less than 3‰, while the relative errors are less than 10 percent.

Simultaneously, a gas chromatograph (GC), used as the reference instrument, was placed in a temporary cabin approximately 50 meters away from the wellhead to ensure safety, as it employs a hydrogen flame ionization detector. The GC system was equipped with a silica capillary column (2 mm in diameter, 4 m in length), with nitrogen as the carrier gas at a flow rate of 30 mL/min. Both the injector and detector temperatures were maintained at 60 °C. The FTIR and GC instruments were connected via pipeline, allowing the gas extracted from the wellhead to first pass through the FTIR system for analysis and then be transferred to the GC for subsequent measurement.

The detection limits of the 11 characteristic gases using the FTIR spectrometer are shown in Table 6.

Furthermore, the indication error of the FTIR analyzer was evaluated based on data collected from two coal mine field sites—Liujia Coal Mine and Fengshuigou Coal Mine in Chifeng City, Inner Mongolia. The GC results served as the reference standard. Since the GC system is capable of quantifying only eight gas species—oxygen (O_2_), nitrogen (N_2_), CH_4_, C_2_H_6_, C_2_H_4_, C_2_H_2_, CO, and CO_2_—the error analysis focused on six of these gases.

As presented in Table 7, both the indication error and relative error of the developed online analyzer for coal spontaneous combustion characteristic gases satisfy the analytical performance criteria established at the outset of the project. Specifically, for gas concentrations ranging from 0 to 3% of the F.S., the indication error remains below 3‰ F.S.; and for concentrations between 3% and 100% F.S., the relative error is maintained under 10%.

These results not only confirm the accuracy and reliability of the proposed analytical instrument in measuring key gases associated with coal spontaneous combustion but also demonstrate its potential for effective early warning and prediction in practical mining applications.

## 4. Conclusions

When using Fourier Transform Infrared (FTIR) spectroscopy to quantitatively analyze alkane gases, the large variety of analytes and severe spectral overlap limit the accuracy and computational efficiency of the quantitative analysis results significantly. Based on the types of gases found in coal mines and their infrared spectral distribution characteristics, this paper proposes a quantitative analysis method for coal mine gases.

The method was validated using standard gas mixtures, and the experimental results show that it can accurately quantify key gases even under spectral interference. Furthermore, the method was applied to real coal mine gas samples, and the results were consistent with reference measurements, confirming its robustness and practical applicability in real-world mining environments. Overall, this framework demonstrates strong potential for enhancing real-time monitoring, early warning capabilities, and safety management in underground coal mining operations.

## Figures and Tables

**Table 1:** The prediction results of alkane gases with a concentration of 500 ppm by IVPA methods.

Evaluation Index	Gas Composition				
	CH_4_	C_2_H_6_	C_3_H_8_	n-C_4_H_10_	i-C_4_H_10_
Predicted concentration (ppm)	−0.54	3.08	−1.84	−5.09	497.43
Cross-sensitivity (%)	0.11	0.62	0.37	1.02	−

**Table 2:** The fitting results obtained using the three different methods (unit: ppm).

Actual Concentration	Quadratic Polynomial	Quartic Polynomial	Third Spline
5	19	5.1	4.9
10	22	9.8	12
20	30	20.2	21
50	52	50.1	48
100	91	101	94
200	178	199	196
500	475	510	512
1000	987	994	992
2000	2073	1995	2003
3000	2959	2994	2999

**Table 3:** The selected spectral lines for each gas.

Gas Name	Selected Spectral Lines (cm^−1^)	Gas Name	Selected Spectral Lines (cm^−1^)
CO_2_	2287, 2360, 2386	C_2_H_2_	730, 805
CO_2_	2029, 2170, 2238	C_3_H_6_	2840, 2920
C_2_H_4_	3205, 3312, 3365	SF_6_	610, 614, 616

**Table 4:** Repeatability test results for gases with distinct absorption peaks (unit: ppm).

Number of Measurements	CO_2_	CO	C_2_H_4_	C_2_H_2_	C_3_H_6_	SF_6_
	20,000	101.5	500	5	100	10.1
1	21,450.6	89.2	499.7	4.1	92.4	9.8
2	21,465.0	90.0	500.0	4.2	93.3	9.5
3	21,641.8	91.9	496.1	3.8	94.7	10.0
4	21,533.9	94.4	500.3	3.9	93.7	9.5
5	21,368.6	93.8	502.1	4.0	95.1	9.8
6	21,456.7	89.1	500.2	3.9	92.4	9.7
7	21,517.7	93.4	501.6	4.0	92.5	9.6
8	21,504.3	92.8	502.3	4.2	94.6	9.9
9	21,472.6	91.7	499.9	3.8	93.8	9.7
10	21,389.1	92.5	501.4	4.1	95.2	9.6
Average value	21,480.0	91.9	500.4	4.0	93.8	9.7
Standard deviation	77.07	1.89	1.78	0.14	1.10	0.17
RSD (%)	0.36	2.05	0.35	3.54	1.18	1.71
Error of indication	+7.4%	−0.01‰F.S.	+0.72%	−0.33‰F.S.	−2.1‰F.S.	−0.13‰F.S.

RSD: relative standard deviation.

**Table 5:** Repeatability test results for gases with overlapping absorption peaks (unit: ppm).

Number of Measurements	CH_4_	C_2_H_6_	C_3_H_8_	n-C_4_H_10_	i-C_4_H_10_
	10,000	1001.2	1000	100	100
1	9851.2	931.3	1041.5	87.6	90.8
2	9845.5	934.1	1048.7	91.1	94.9
3	9856.3	926.6	1055.2	89.3	96.1
4	9851.2	931.2	1054.4	88.6	93.0
5	9852.4	925.5	1064.6	94.2	92.6
6	9856.9	923.3	1045.1	90.5	95.1
7	9843.8	927.8	1047.3	87.4	96.3
8	9847.0	924.9	1052.5	91.8	95.5
9	9854.1	931.5	1043.8	89.7	91.9
10	9855.7	932.6	1049.3	91.0	89.4
Average value	9851.4	929.9	1050.2	90.1	92.8
Standard deviation	4.63	3.37	6.74	2.06	2.21
RSD (%)	0.05	0.36	0.64	2.29	2.38
Error of indication	−1.49%	−7.1%	+5.0%	−2.0‰F.S.	−1.4‰F.S.

RSD: relative standard deviation.

**Table 6:** Detection limits of the 11 characteristic gases (unit: ppm).

Gas Name	Detection Limits	Gas Name	Detection Limits
CH_4_	0.5	C_2_H_2_	0.2
C_2_H_6_	1	C_3_H_6_	0.5
C_3_H_8_	0.5	CO	1
i-C_4_H_10_	0.5	CO_2_	0.5
n-C_4_H_10_	0.5	SF_6_	0.2
C_2_H_4_	0.5		

**Table 7:** Indication errors of the analysis results.

**Gas Location**	**CH_4_**	**C_2_H_6_**	**C_2_H_4_**	**C_2_H_2_**	**CO**	**CO_2_**
1	−0.072‰F.S.	−0.330‰F.S.	0.483‰F.S.	0.108‰F.S.	−0.015‰F.S.	−0.193‰F.S.
2	−0.014‰F.S.	−0.215‰F.S.	−0.150‰F.S.	0.033‰F.S.	−0.014‰F.S.	−0.085‰F.S.
3	−0.015‰F.S.	−0.370‰F.S.	0.375‰F.S.	0.073‰F.S.	−0.012‰F.S.	−0.111‰F.S.
4	−5.39%	−4.14%	0.683‰F.S.	−0.083‰F.S.	−0.014‰F.S.	−0.27%
5	−0.026‰F.S.	−0.190‰F.S.	0.867‰F.S.	0.150‰F.S.	0.001‰F.S.	−0.053‰F.S.

## Data Availability

The original contributions presented in this study are included in the article. Further inquiries can be directed to the corresponding author(s).

## References

[1] Wojtacha-Rychter K., Smoliński A. (2019). Selective adsorption of ethane, ethylene, propane, and propylene in flammable gas mixtures on different coal samples and implications for fire hazard assessments. Int. J. Coal Geol..

[2] Liang Y., Tian F., Feng W., Shao Z., Meng X., Chen C. (2021). Research progress of coal mine gas detection technology in China. China Coal Soc..

[3] Zhao Z., Liang Y., Jin L., Song S., Wang J., Wang J. (2022). Research progress of FTIR detection technology and its application in coal mine. Saf. Coal Mines..

[4] Loof D., Thüringer O., Zielasek V., Pranti A.S., Lang W., Bäumer M. (2024). In-operando FTIR study of ligand-linked Pt nanoparticle networks employed as catalysts in hydrogen gas micro sensors. Nanoscale Adv..

[5] Li X., Cao Z., Xu Y. (2025). Characteristics and trends of coal mine safety development. Energy Sources Part A.

[6] Peng Y., Yang L., Ju X., Liao B., Ye K., Li L., Cao B., Ni Y. (2020). A comprehensive investigation on the thermal and toxic hazards of large format lithium-ion batteries with LiFePO4 cathode. J. Hazard. Mater..

[7] Fahelelbom K.M., Saleh A., Al-Tabakha M.M., Ashames A.A. (2022). Recent applications of quantitative analytical FTIR spectroscopy in pharmaceutical, biomedical, and clinical fields: A brief review. Rev. Anal. Chem..

[8] Ye Q., Meng X. (2022). Highly efficient authentication of edible oils by FTIR spectroscopy coupled with chemometrics. Food Chem..

[9] Hutchinson G., Welsh C.D., Burés J. (2020). Use of standard addition to quantify in situ FTIR reaction data. J. Org. Chem..

[10] Zhao F., Yan W.J. (2025). Monitoring and prevention of gas explosions in underground coal mines using a co-prototype design model for dynamic disaster response. Sci. Rep..

[11] Shuai W., Wu X., Chen C., Zuo E., Chen X., Li Z., Lv X., Wu L., Chen C. (2024). Rapid diagnosis of rheumatoid arthritis and ankylosing spondylitis based on Fourier transform infrared spectroscopy and deep learning. Photodiagn. Photodyn. Ther..

[12] Berisha S., Lotfollahi M., Jahanipour J., Gurcan I., Walsh M., Bhargava R., Nguyen H.V., Mayerich D. (2019). Deep learning for FTIR histology: Leveraging spatial and spectral features with convolutional neural networks. Analyst.

[13] Li L., Zhang Q., Yuan X., Yang H., Qin S., Hong L., Pu L., Li L., Zhang P., Zhang J. (2024). Study of the molecular structure of proteins in fermented Maize-Soybean meal-based rations based on FTIR spectroscopy. Food Chem..

[14] Zhao A., Tang X., Wang E., Zhang Z., Liu J. (2013). Quantitative Analysis of Transformer Oil Dissolved Gases Using FTIR. Spectrosc. Spectr. Anal..

[15] Shaltout A.A., Seoudi R., Almalawi D.R., Abdellatief M., Tanthanuch W. (2024). Quantitative phase analysis and molecular structure of human gallstones using synchrotron radiation X-ray diffraction and FTIR spectroscopy. Spectrochim. Acta Part A.

[16] Goldschmidt J., Nitzsche L., Wolf S., Lambrecht A., Wöllenstein J. (2022). Rapid quantitative analysis of IR absorption spectra for trace gas detection by artificial neural networks trained with synthetic data. Sensors.

[17] Tang X., Li Y., Zhu L., Zhao A., Liu J. (2015). On-line multi-component alkane mixture quantitative analysis using Fourier transform infrared spectrometer. Chemom. Intell. Lab. Syst..

[18] Shoukat U., Baumeister E., Knuutila H.K. (2019). ATR-FTIR model development and verification for qualitative and quantitative analysis in MDEA–H_2_O–MEG/TEG–CO_2_ blends. Energies.

[19] Li Z., Pang W., Liang H., Chen G., Zheng X., Ni P. (2022). Multicomponent Alkane IR measurement system based on dynamic adaptive moving window PLS. IEEE Trans. Instrum. Meas.

[20] Zhang F., Tang X., Tong A., Wang B., Wang J., Lv Y., Tang C., Wang J. (2020). Baseline correction for infrared spectra using adaptive smoothness parameter penalized least squares method. Spectrosc. Lett..

[21] Zhang F., Tang X., Tong A., Wnag B., Tang C., Wang J. (2021). A Mid-Infrared Wavelength Selection Method Based on the Impact Value of Variables and Population Analysis. Spectrosc. Spectr. Anal..

[22] Gianella M., Sigrist M.W. (2009). Improved algorithm for quantitative analyses of infrared spectra of multicomponent gas mixtures with unknown compositions. Appl. Spectrosc..

[23] Tanabe A., Morita S., Tanaka M., Ozaki Y. (2008). Multivariate curve resolution analysis on the multi-component water sorption process into a poly(2-methoxyethyl acrylate)film. Appl. Spectros..

[24] Duan X., Wang M. (2019). Quantitative Analysis of Multi-Component Gases in Underground by Improved PSO-SVM Algorithm. Spectrosc. Spectr. Anal..

[25] Yang H., Griffiths P.R., Tate J.D. (2003). Comparison of partial least square regression and multi-layer networks for quantification of nonlinear systems and application to gas phase Fourier transform infrared spectra. Anal. Chim. Acta.

